# Application of RNA sequencing to understand the response of rice seedlings to salt-alkali stress

**DOI:** 10.1186/s12864-023-09121-x

**Published:** 2023-01-14

**Authors:** Xiaoning Ren, Jiahui Fan, Xin Li, Yu Shan, Lanlan Wang, Lianju Ma, Yueying Li, Xuemei Li

**Affiliations:** College of Life Science, Shenyang Normal University, 110034 Shenyang, China

**Keywords:** Bioinformatic, *Oryza sativa* L., RNA-seq, Salt-alkali stress

## Abstract

**Background:**

Salt-alkali stress represents one of the most stressful events with deleterious consequences for plant growth and crop productivity. Despite studies focusing on the effects of salt-alkali stress on morphology and physiology, its molecular mechanisms remain unclear. Here, we employed RNA-sequencing (RNA-seq) to understand how Na_2_CO_3_ stress inhibits rice seedling growth.

**Results:**

Na_2_CO_3_ stress significantly inhibited the growth of rice seedlings. Through RNA-seq, many differentially expressed genes (DEGs) were shown to be potentially involved in the rice seedling response to salt-alkali stress. After 1-day and 5-day treatments, RNA-seq identified 1780 and 2315 DEGs in the Na_2_CO_3_-treated versus -untreated rice seedling shoots, respectively. According to the gene ontology enrichment and the Kyoto Encylopedia of Genes and Genomes annotation of DEGs, the growth-inhibition processes associated with salt-alkali stress involve a myriad of molecular events, including biosynthesis and metabolism, enzyme activity, and binding, etc.

**Conclusion:**

Collectively, the transcriptome analyses in the present work revealed several potential key regulators of plant response to salt-alkali stress, and might pave a way to improve salt-alkali stress tolerance in rice.

**Supplementary Information:**

The online version contains supplementary material available at 10.1186/s12864-023-09121-x.

## Background

Salt-alkali stress has been emerging as a severe threat to the plant growth and crop productivity. It is reported that there are more than 1 billion hm^2^ salinization-alkalization lands worldwide [1]. Soil salinization and alkalization can produce a multitude of harmful effects on plants, including osmotic pressure, high pH stress and disrupt ionic balance [2, 3], thereby impeding plant growth, development and yield [4, 5].

Rice represents the second most crucial cereal after the wheat [6], and provides the primary source of calorie to a large fraction population globally [7–9]. However, rice displays poor salt-alkali resistance, especially at the early seedling stage [10, 11]. Previous researches have demonstrated that the rice seedlings under salt-alkali stress grew slowly and suffered from a significant decrease in chlorophyll content, cell membrane stability, and relative water content (RWC) [12]. Moreover, our previous work established that salt-alkali stress led to a series of functional abnormalities in rice seedlings, including photosynthetic capacity, ROS equilibrium, antioxidant system, organic acid and mineral element metabolism [13–15]. However, the definite molecular events of rice seedling response to salt-alkali stress are largely unknown.

At present, advances in molecular/omics/sequencing technology have been opening new paths for investigating the impacts of environmental stress on plants, and RNA-sequencing (RNA-seq) is one of the rapid development technologies [16]. RNA-seq has been used extensively due to its ability to provide efficient, rapid, and comprehensive transcript information [17]. A previous study reported that comparative transcriptomic analysis of two *Vicia sativa* L. cultivars could reveal the crucial role of metal transporters in cadmium tolerance [18]. Baldoni et al. [19] applied RNA-seq for investigating major differences in the root early responses to osmotic stress. A study by Xu and colleagues [20] discovered the molecular mechanisms behind cotton response to salt stress through a comprehensive transcriptome analysis. In the present work, we focused on the molecular players involved in rice seedling response to salt-alkali stress by utilizing RNA-seq. Our findings provide the theoretical basis for crop response to salt-alkali stress.

## Results

### Phenotype and growth parameters

**Fig. 1:**
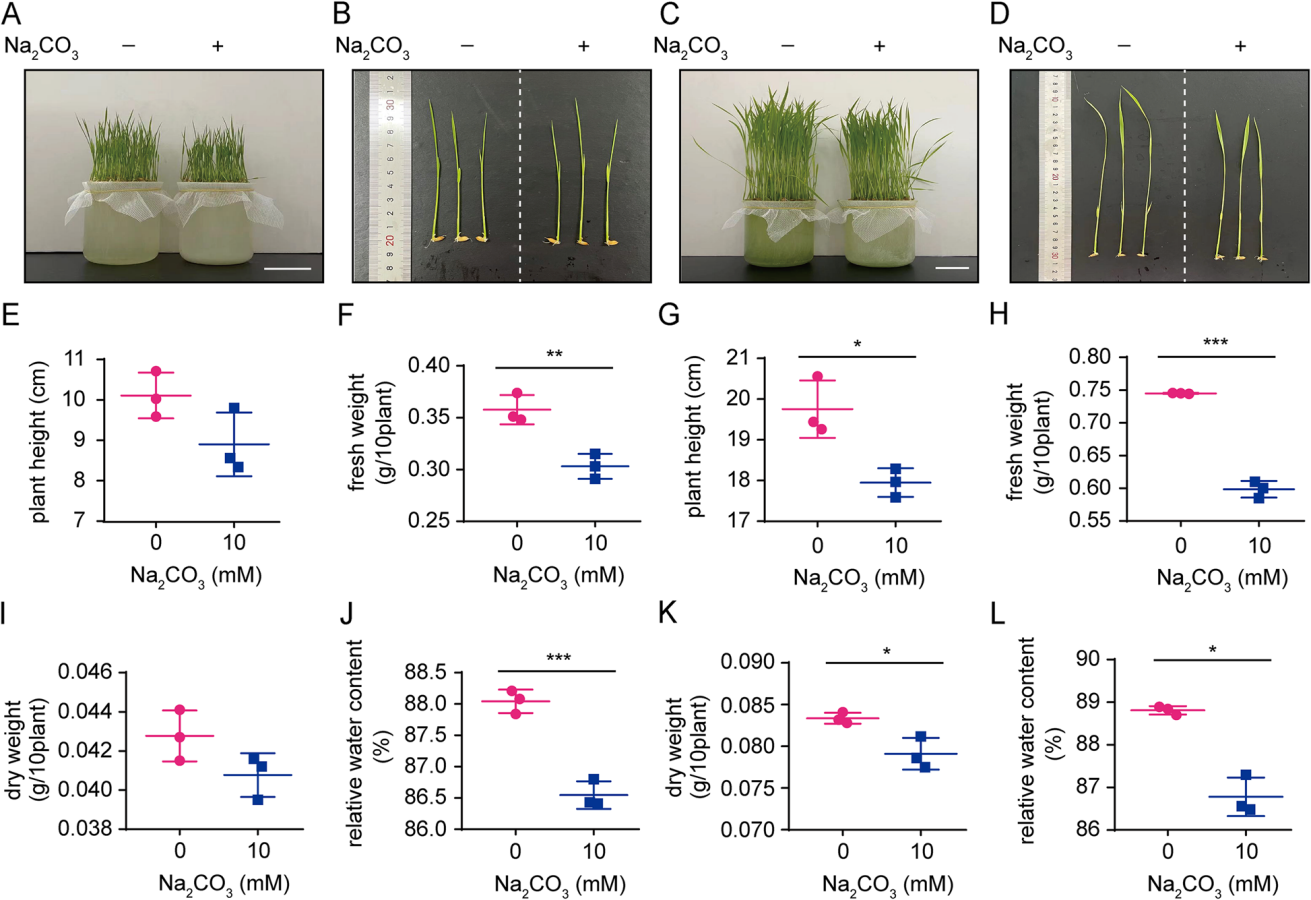
The growth of Na_2_CO_3_-treated (N+) versus -untreated (N-) rice seedlings. Gross inspection of N + versus N- rice seedling shoots at day 1 (**A**, **B**), day 5 (**C**, **D**). Statistical analysis for plant height (**E**), fresh weight (**F**), dry weight (**I**) and relative water content (**J**) from N + versus N- rice seedling shoots at day 1. Statistical analysis for plant height (**G**), fresh weight (**H**), dry weight (**K**) and relative water content (**L**) from N + versus N- rice seedling shoots at day 5

In response to Na_2_CO_3_ treatment, fresh weight and RWC of rice seedling shoots presented a significant inhibition (Fig. 1A, B, F, J), while plant height and dry weight did not change significantly at day 1 (Fig. 1E, I). The growth-inhibition in rice seedlings was more pronounced upon 5 d of Na_2_CO_3_ treatment (Fig. 1C, D, G, H, K, L). These findings confirmed an adverse effect of salt-alkali stress on plants.

### General transcriptomic profiling

**Fig. 2:**
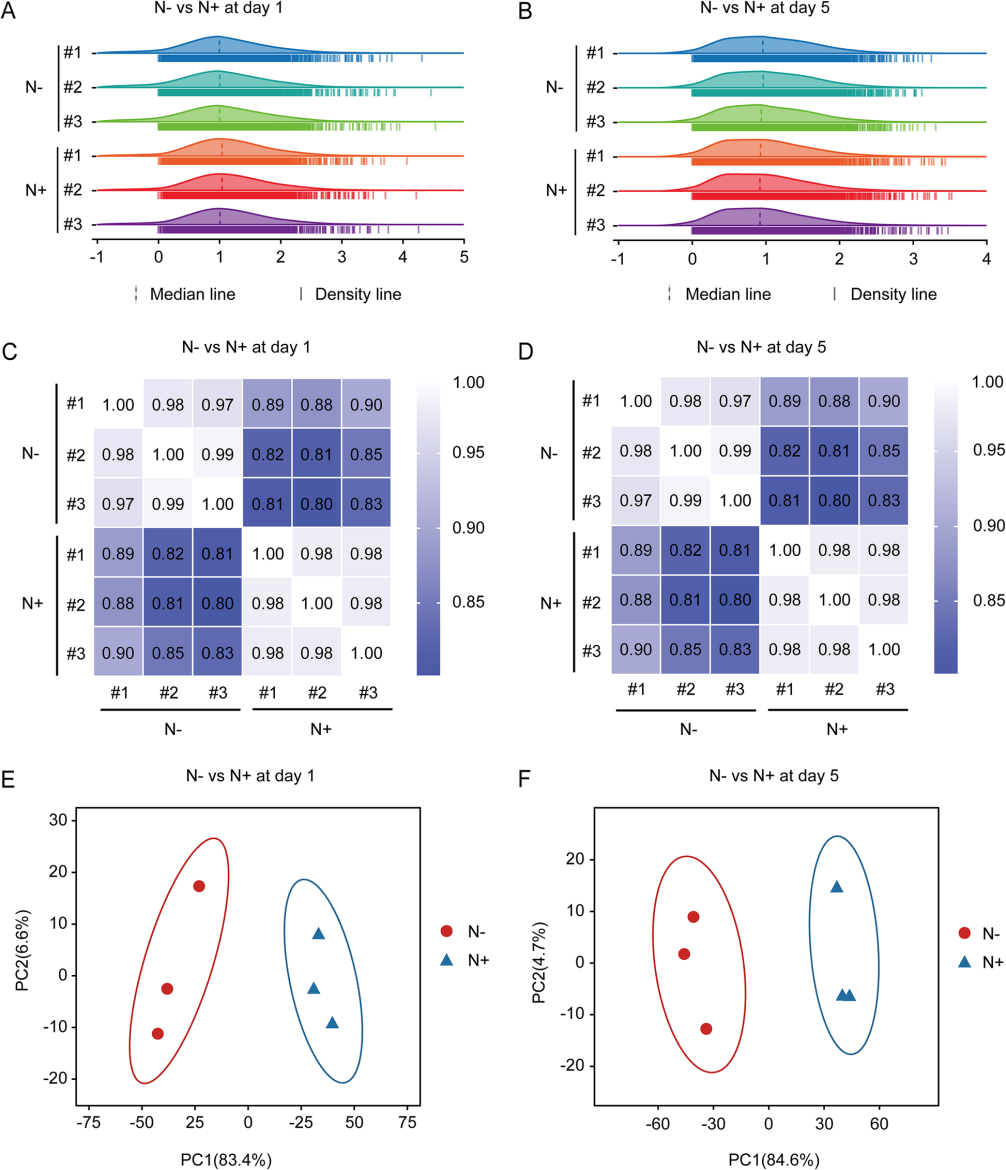
RNA-seq results for Na_2_CO_3_-treated (N+) versus -untreated (N-) rice seedling shoots. Ridgeline plots, principal component analysis (PCA) and heatmaps using DEGs obtained from N + versus N- shoots at day 1 (**A**, **C**, **E**) and day 5 (**B**, **D**, **F**)

After filtering low-quality reads and trimming adapters, approximately 88 gigabases of clean reads were used for assembly and analysis, and the Q30 base percentage was greater than 93.87% (Supplementary material 1: Table S1). The ratio mapped to the *japonica* genome were 95.24-95.94% (Supplementary material 1: Table S2). After 1-day or 5-day treatment, differential expression analysis was performed in the Na_2_CO_3_-treated versus -untreated rice seedling shoots. Results showed compared with 1-day treatment, the 5-day Na_2_CO_3_ treatment elicited a greater number of DEGs (Fig. 2A, B). Spearman correlation and principal component analysis demonstrated good similarity of biological replicates under the same condition, and discriminated samples with different treatments (Fig. 2C, D, E, F). Additionally, 6 up-regulated and 4 down-regulated genes were randomly selected to validate RNA-seq accuracy through qRT-PCR. Results showed that the RNA-seq data matched well with the RNA-seq qRT-PCR findings (Supplementary material 1: Fig. S1, Table S3). Taken together, we concluded that the RNA-seq could reliably identify genes that participated in rice seedling response to Na_2_CO_3_ treatment.

### DEGs evaluation and bioinformatic analysis in Na_2_CO_3_ treatment on day 1

**Fig. 3:**
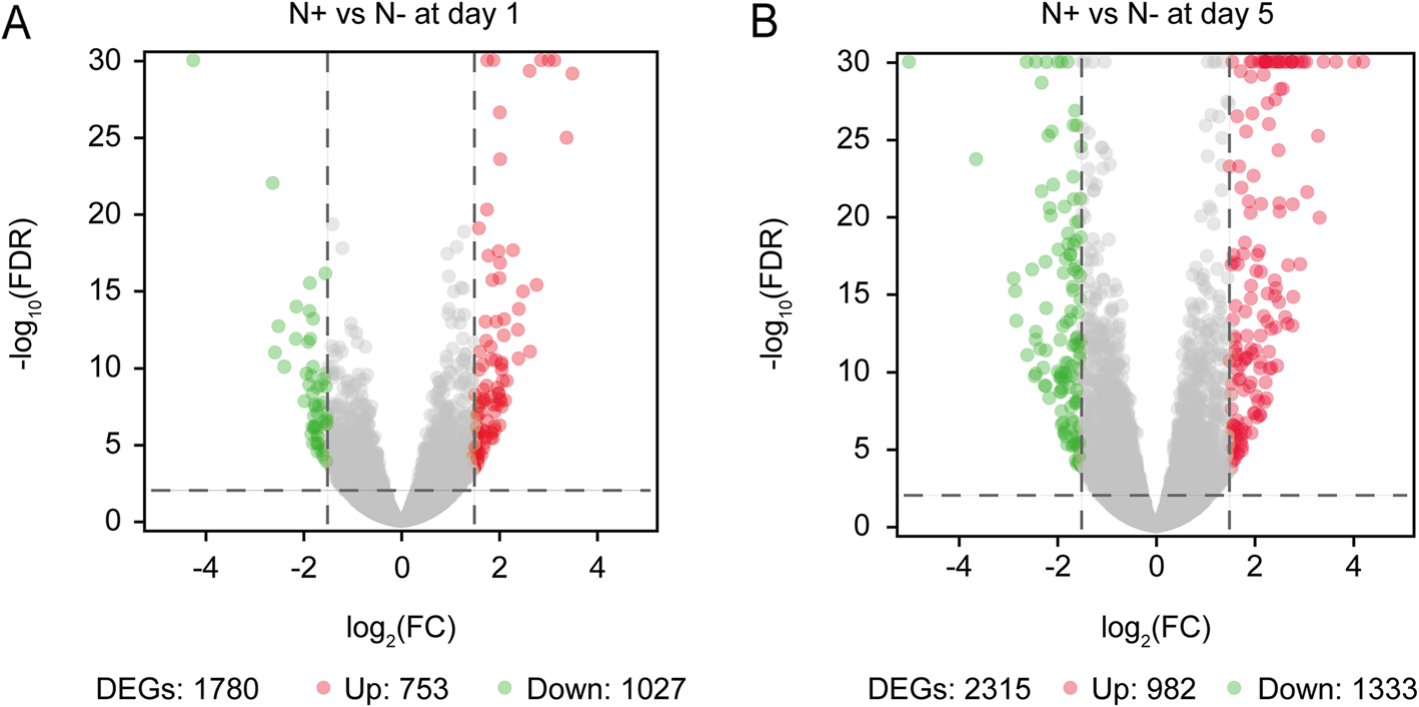
Volcano plots of DEGs in Na_2_CO_3_-treated (N+) versus -untreated (N-) shoots at day 1 (**A**) and day 5 (**B**). Significantly up- and down-regulated genes were represented by red and green dots, respectively

**Fig. 4:**
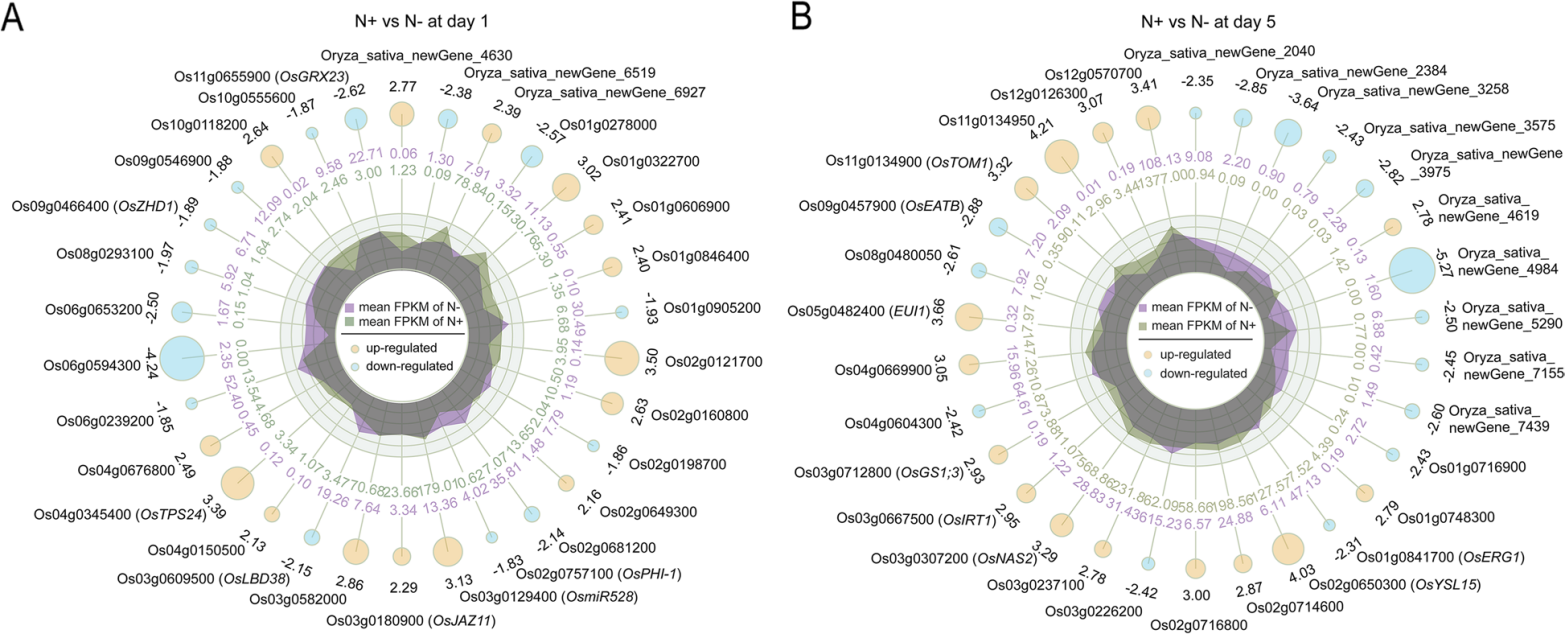
Top 15 up- (yellow) and 15 down-regulated (blue) genes in Na_2_CO_3_-treated (N+) versus -untreated (N-) shoots at day 1 (**A**) and day 5 (**B**). The first lap indicates the names of 15 top up- and down-regulated genes. The second lap indicates the fold change in N + versus N- rice seedling shoots. The third lap indicates larger circle presented larger fold change. The fourth and fifth lap show the mean FPKM of N- and N+, respectively

**Fig. 5:**
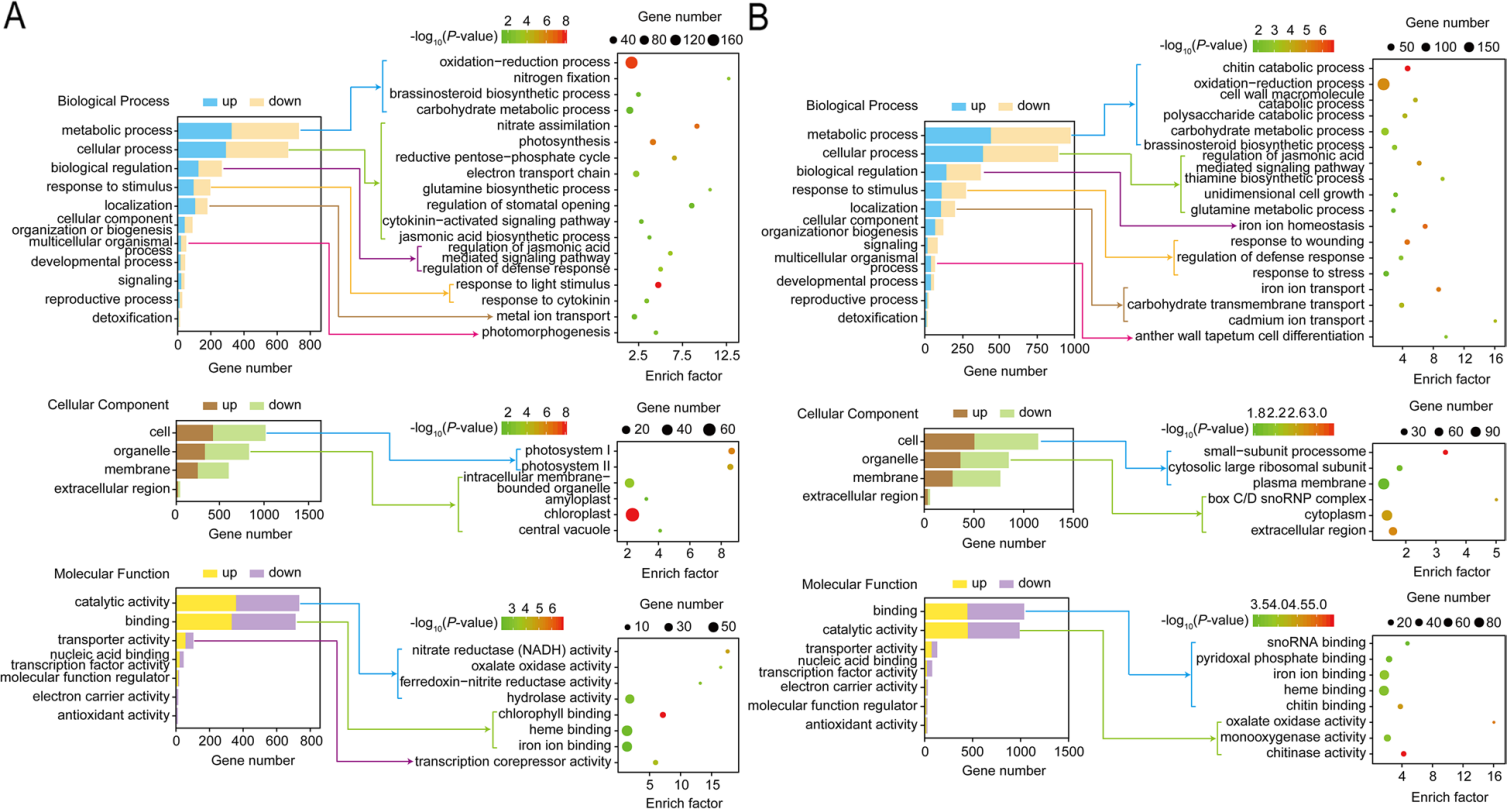
GO enrichment analysis for DEGs in Na_2_CO_3_-treated (N+) versus -untreated (N-) rice seedling shoots at day 1 (**A**) and day 5 (**B**)

**Fig. 6:**
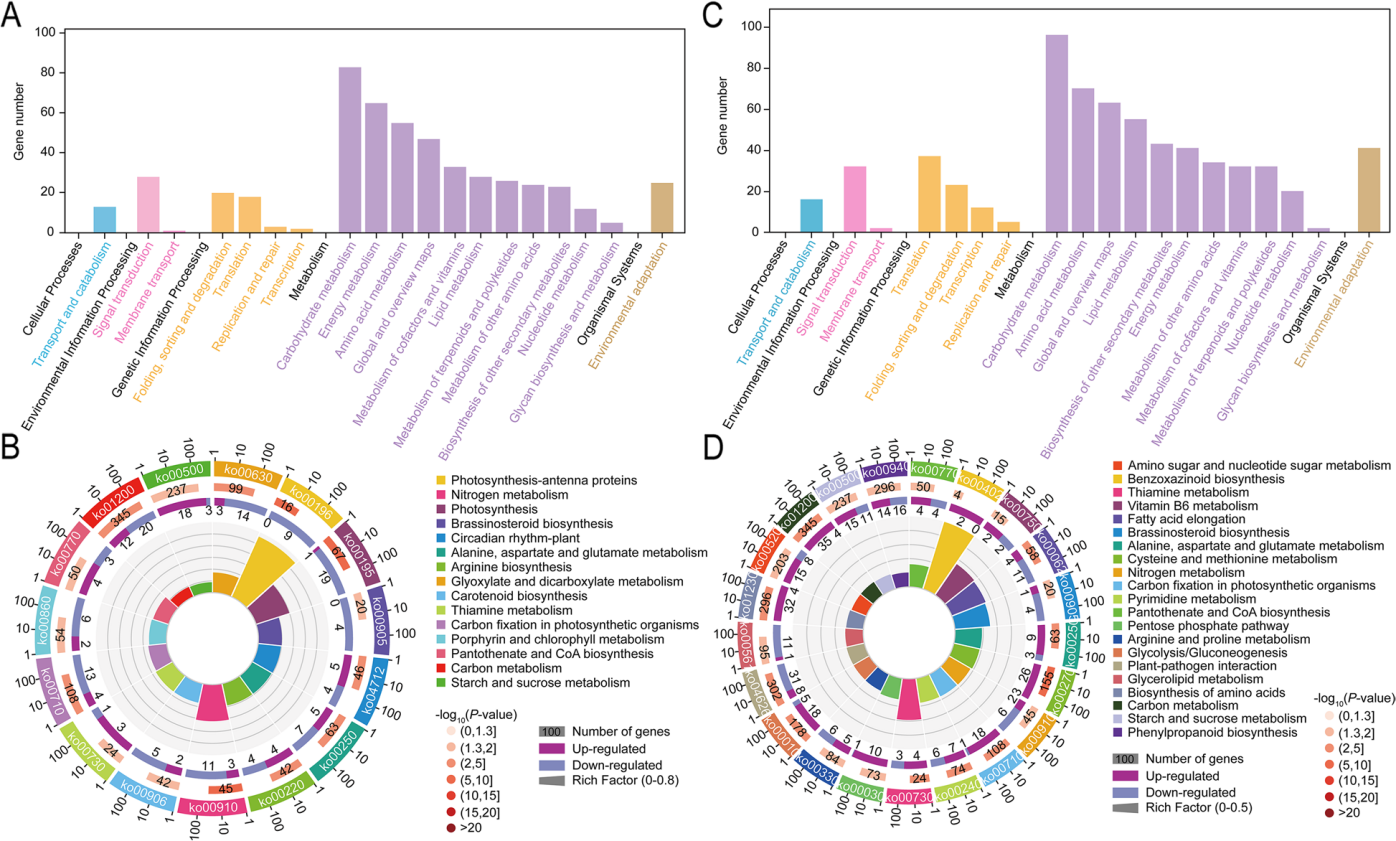
KEGG enrichment analysis for DEGs in Na_2_CO_3_-treated (N+) versus -untreated (N-) rice seedling shoots at day 1 (**A**, **B**) and day 5 (**C**, **D**). **A**, **C** DEGs enrichment in KEGG related to cellular processes, environmental information processing, genetic information processing, metabolism and organismal systems. **B**, **D** The significantly enriched KEGG pathways

A total of 1780 DEGs were identified upon one day of Na_2_CO_3_ treatment in the rice seedling shoots. Among these genes, 753 up-regulated and 1027 down-regulated genes were found (Fig. 3A, Supplementary material 2: Table S4). The top 15 up- and down-regulated genes were presented in Fig. 4A. According to the gene ontology (GO) enrichment annotation of the 1780 DEGs, BP terms showed that the DEGs were enriched mainly in metabolic process, cellular process, biological regulation and response to stimulus, specifically, in oxidation-reduction process, carbohydrate metabolic process, photosynthesis, and response to light stimulus. Among CC terms, the process of cell, organelle and membrane was enriched by 1015, 830 and 602 DEGs, respectively. Out of MF terms, the process of catalytic activity and binding had the most abundant functions; in detail, the DEGs were involved in heme binding, iron ion binding and hydrolase activity (Fig. 5A). Based on the Kyoto Encylopedia of Genes and Genomes (KEGG) enrichment annotation of the 1780 DEGs, 15 pathways were significantly enriched such as photosynthesis (ko00195), nitrogen metabolism (ko00910), glyoxylate and dicarboxylate metabolism (ko00630), carbon metabolism (ko01200) and carotenoid biosynthesis (ko00906), etc. (Fig. 6A, B).

### DEGs evaluation and bioinformatic analysis in Na_2_CO_3_ treatment on day 5

In the Na_2_CO_3_-treated versus -untreated rice seedling shoots, long-term stress (5 d treatment) resulted in 2315 DEGs, including 982 up- and 1333 down-regulated genes (Fig. 3B, Supplementary material 2: Table S5). The top 15 up- and down-regulated genes were displayed in Fig. 4B. On the basis of 2315 DEGs, GO enrichment annotation was performed. The significantly enriched BP terms mainly included metabolic process, cellular process, biological regulation, response to stimulus, specifically, in chitin-catabolic process, oxidation-reduction process, carbohydrate metabolic process, regulation of jasmonic acid mediated signaling pathway, iron ion homeostasis. Among CC terms, the number of DEGs enriched in the process of cell, organelle and membrane was greatest, especially in plasma membrane and cytoplasm. Out of MF terms, catalytic activity and binding had the most abundant functions; in detail, the DEGs took effects in iron ion binding, heme binding and monooxygenase activity (Fig. 5B). KEGG enrichment annotation of 2315 DEGs demonstrated that 454 DEGs were enriched in 110 pathways. The significantly enriched KEGG pathways also included carbon fixation in photosynthetic organisms (ko00710), plant-pathogen interaction (ko04626), carbon metabolism (ko01200), nitrogen metabolism (ko00910), vitamin B6 metabolism (ko00750), starch and sucrose metabolism (ko00500), etc. (Fig. 6C, D).

### Overlapped DEGs, GO terms and pathways between the 1 d and 5 d treatment

A total of 405 overlapped DEGs associated with rice seedling response to Na_2_CO_3_ treatment were identified between the 1 d and 5 d treatment (Supplementary material 1: Fig. S2A). Among these genes, 341 genes (175 up-regulated and 166 down-regulated) displayed the same changing pattern (Supplementary material 1: Table S6, S7). A further increased expression in response to 5 d versus 1 d treatment was found in 69 of the 175 up-regulated genes (Supplementary material 1: Table S6 ). Among these 166 down-regulated genes, the expression levels of 72 genes were further decreased upon 5 d versus 1 d treatment (Supplementary material 1: Table S7). These findings suggested long-term Na_2_CO_3_ treatment enhanced the effects on the genes. In addition, 64 DEGs had an opposite expression pattern between the 1 d and 5 d treatment (Supplementary material 1: Fig. S2B); that is, 48 genes were up-regulated at day 1 and down-regulated at day 5; 16 genes were down-regulated at day 1 and up-regulated at day 5. These genes might play pleiotropic roles in rice response to salt-alkali stress.

In addition, the short-term and long-term salt-alkali stress shared 39 common GO terms (Supplementary material 1: Table S8) and 8 KEGG pathways (Supplementary material 1: Table S9), including oxalate oxidase activity (GO:0050162), oxidation-reduction process (GO:0055114), thiamine biosynthetic process (GO:0009228), brassinosteroid biosynthetic process (GO:0016132), etc.

## Discussion

Crops are often subjected to severe abiotic stress such as water deficit [21–23], salinity [24–26], heavy metal exposure [27–29], etc. Thus, understanding how plants respond to these stresses is helpful to improve crop productivity. Our findings here and previous studies [13] confirmed the inhibition of growth of rice seedlings under salt-alkali stress. In this work, we used RNA-seq to investigate the molecular players behind rice seedling response to salt-alkali stress.

Salt-alkali stress-induced plant responses are orchestrated by a complex network of cross-talk between signaling pathways and sensors. This work demonstrated that salt-alkali stress caused many DEGs associated with oxalate oxidase (OxO) that catalyzes the oxidative breakdown of oxalate to H_2_O_2_ and CO_2_ [30]. Previous studies reported that abiotic stresses of salinity [31], heat, and heavy metal ions [32] could increase *OxO* gene expression and/or *OxO* activation. In line with these results, this work found that *OsOxO1*, *OsOxO3*, and *OsOxO4* were up-regulated upon 1 d and 5 d treatment. Previous work demonstrated an increase in H_2_O_2_ content in response to salt-alkali stress [15]. In agreement, we found that salt-alkali stress elicited an increase in the expression of *OsOxO1*, *OsOxO3* and *OsOxO4* that participated in H_2_O_2_ metabolism.

Glutaredoxins (*GRXs*) are a class of oxidoreductase which are reduced by glutathione [33, 34], and exert various functions in plants, such as regulation of [Fe-S] assembly, Krebs cycle, Calvin cycle and signaling pathway, plant response to phytohormones, etc. [35]. Previous studies demonstrated silencing the expression of *GRXs* genes in plants reduced tolerance to abiotic stress, whereas overexpression of *GRXs* genes exhibited an opposite function [36–38]. In line with this, we identified 4 down-regulated *GRX* genes (*OsGRX23*, *OsGRX24*, *OsGRX28*, *OsGRX29*) might be involved in rice seedling response to Na_2_CO_3_ treatment. These findings were further confirmed by a study by Garg and co-workers [33]. Given the functions of *GRXs* genes in plants, we concluded that down-regulation of *OsGRX23*, *OsGRX24*, *OsGRX28*, *OsGRX29* might contribute to the rice sensitivity to salt-alkali stress.

Thiamine is an important factor in the activity of several enzymes associated with major metabolic pathways, including the Krebs cycle, the pentose phosphate pathway, the branched-chain amino acid pathway, anaerobic respiration, and pigment biosynthesis [39]. Moreover, thiamine was found to correlate with disease resistance in plants [39]. The present work indicated that 3 genes (Os03g0679700, Os07g0190000, *OsXNP*) associated with plant response to salt-alkali stress were enriched in the thiamine biosynthetic process. These genes are associated with thiamine metabolism, which might contribute to rice seedling response to salt-alkali stress.

Brassinosteroid (BRs) are steroid hormones that are essential for plant growth and development. These hormones are able to regulate cell division, cell elongation, xylem differentiation, reproduction, photomorphogenesis and stress response [40, 41]. Accordingly, abnormalities in genes encoding the main components of the BR synthesis and signaling pathways could result in severe dwarfism, impaired organ growth and development, and limited plant fertility and yield [41]. This study demonstrated that 4 genes correlating with rice seedling response to salt-alkali stress were enriched in the brassinosteroid biosynthetic process. Among these 4 genes, 3 genes (Os12g0139300, Os11g0143200, *OsABA8ox1*) and 1 gene (Os07g0519600) were down-regulated and up-regulated in both 1 d and 5 d treatment, respectively. These 4 DEGs maybe play key roles in the growth limitation of rice seedlings in response to salt-alkali stress.

Photosynthesis is a crucial biological process which could often be influenced by abiotic stress [42]. Our previous work reported that Na_2_CO_3_ stress could result in significant decrease in chlorophylls and carotenoid contents in rice under [13]. Furthermore, the present work showed 28 DEGs at day 1 were enriched in photosynthesis, and all genes were down-regulated except Os07g0147900 encoding putative encoding ferredoxin-NADP reductase. Out of these 28 genes, the deletion of *YGL8* [43], *LYL1* [44], *chl9* [45], *OsFdC2* [46] or *OsFd1* [47], was clearly found to decrease the chlorophylls content, thereby resulting in leaves yellow. Furthermore, it is worth mentioning that Os07g0147900 and Os01g0934400 were up- and down-regulated at day 5, and 10 DEGs were enriched in photosynthesis on day 5. Out of the 10 genes, *OsPS1-F* deletion was found to promote yellow-green leaves [48]. Therefore, we concluded that these dysregulated genes might be involved in the regulation of chlorophyll content and photosynthesis.

Because significant responses to salt-alkali stress were detected in the above-ground tissues, this study used the shoots to understand the response of rice seedlings to salt-alkali stress. Our findings suggested that the growth-inhibition of rice seedling following Na_2_CO_3_ treatment might be a cumulative outcome of differential expression of genes. Further clarification of the dynamic process of Na_2_CO_3_ treatment in rice seedlings is the footing stone to determine whether the affected expression of genes in the above-ground tissues is a direct result of Na_2_CO_3_ treatment, or a subsequent consequence of the effects of Na_2_CO_3_ treatment on the below-ground tissues. Given the global changes in the transcriptome in response to abiotic stress [49, 50], there is likely to have genes with similar or opposite expression pattern in the shoots versus roots. It is of great interest to investigate that the genes have similar or different roles in the shoots and roots.

According to our current results, gene expression change in response to salt-alkali stress could be seen in the above-ground tissues of rice seedlings. In line with our previous descriptions [10, 15], the rice seedlings are sensitive to salt-alkali stress, as demonstrated by the findings that 10mM Na_2_CO_3_ treatment could lead to a significant change in their phenotypes and growth parameters. Yet it remains unclear whether 10mM Na_2_CO_3_ treatment could have effects on the salt-alkali tolerant rice variety. Since different rice varieties might have different responses, whether the gene changes in response to salt-alkali stress is unique for salt-alkali sensitive rice variety remains an important knowledge gap in our understanding of this area. Furthermore, although the one and five days of Na_2_CO_3_ treatments were used in this study, further efforts are needed to show whether the two time points could truly reflect the short-term and long-term responses to salt-alkali stress.

At the two sampled time points, the rice seedling shoots were found to have 341 DEGs with the same expression pattern. Of additional interest, our experimental data demonstrated that Na_2_CO_3_ at the two post-treatment sampling time points resulted in 64 DEGs with an opposite expression change. This pattern suggested that the growth-inhibition processes of Na_2_CO_3_ treatment were dynamic and discriminating. Different genes might play similar roles in one specific system, and one gene might take effect in different systems. The functions of these genes are important points in future studies that focus on the molecular level responses of rice to salt-alkali stress.

This work used transcriptomic analysis to identify molecular players related to the responses of rice seedlings to salt-alkaline stress. This is an area where essentially descriptive work might still be needed to serve as a basis for more theoretical/ explanatory work by others. We assume that the “forest” is how growth-inhibition of rice seedlings occurs under salt-alkaline stress, and thus this work tried to uncover the “forest” by RNA-seq. The data contributes to advance the understanding of the abiotic stress-plant interactions by identifying many genes and their functions that are potentially involved in the growth-inhibitory responses of rice seedlings to salt-alkaline stress. This is a study about “trees” with some insights into the “forest”. The biochemical or physiological features from RNA-seq data need to be confirmed by future studies.

## Conclusion

Understanding the molecular mechanism whereby crops respond to salt-alkali stress is an important step to increase the salt-alkali tolerance of plants. This study presents an overview of rice seedling response to salt-alkali stress. Our data provide a solid foundation for future studies to understand the molecular mechanisms underlying the response to salt-alkali stress in plants.

## Materials and methods

### Rice materials and treatment

After hybridizing Liaojing 454 with Shennong 9017, the cultivar Liaoxing NO.1 belonging to *japonica* subspecies was obtained by Liaoning Provincial Crop Variety Certification Committee. Based on the identified effects of salt-alkali stress on Liaoxing NO.1 [13–15], this cultivar was selected for measuring growth indexes and transcriptome profiling. After surface-sterilization, rice seeds were imbibed in deionized water (28 °C/ 24 h), and were transferred to filter paper which had been moistened by deionized water for germinating (30 °C/ 24 h). Subsequently, the germinated seeds were cultivated in 500 ml beaker which contained Hoagland solution in a growth chamber (80% relative humidity, 16 h light 10,000 lx at 28 °C and 8 h dark at 26 °C). Based on our previous findings [51], Na_2_CO_3_-treated rice seedlings were chosen for the transcriptomic analysis on day 1 and day 5 after the treatments in the present study. After 4 d of growth, rice seedlings were subjected to 0 and 10 mM Na_2_CO_3_ treatment. Fresh shoot samples were collected after 1 d and 5 d treatments, respectively. Each treatment was repeated for three times.

### Analysis of growth indexes

After exposure to 1 d and 5 d Na_2_CO_3_ treatment, rice seedlings were collected to measure shoot length, fresh weight (FW) and dry weight (DW). Dry weight was determined after drying them at 80 °C for 12 h. RWC = (FW-DW) ×100/FW [52, 53].

### RNA extraction and sequencing

At similar time points after 1 d and 5 d of Na_2_CO_3_ treatment, rice seedling shoots were collected, immediately frozen and stored at -80 °C for subsequent RNA extraction. Total RNA was extracted using the RNA isolation Kit RN40 (Aidlab Bio Co Ltd, Beijing). The purity and concentration of extracted RNA were checked on a NanoDrop 2000 (Thermo Fisher Scientific, Wilmington, DE), and the RNA integrity was verified by the Agilent Bioanalyzer 2100 system (Agilent Technologies, CA, USA). The preparation of cDNA library was done using quality-controlled RNA samples and then was subjected to sequencing on HiSeq 2500 (Illumina, CA, USA).

### Data processing and bioinformatic analysis

Low-quality bases and adapter sequences were removed from the raw data, and high-quality data of RNA-seq were mapped to the *japonica* reference genome (https://rapdb.dna.affrc.go.jp/) using HISAT2 (version 2.0.4). Transcript expression levels were estimated using fragments per kilobase per million reads (FPKM) values that were calculated according to the previously described formula [54, 55]. Based on the FPKM values, differential expression analysis was performed using the DESeq2 (version 1.6.3), and genes showing fold changes (FC) ≥ 1.5 with a false discovery rate (FDR) ≤ 0.01 were considered to be differentially expressed.

Gene Ontology analysis was performed using the GOseq R packages for the enrichment analysis. The functional annotation of DEGs was reflected in three major GO classification: Cellular component (CC), Biological process (BP), and Molecular function (MF). Kyoto Encyclopedia of Genes and Genomes (KEGG) pathways were conducted to identify the biological pathways associated with rice seedling response to Na_2_CO_3_ stress. The statistical test for GO and KEGG analysis was Fisher’s Exact, and *P* values < 0.05 were considered to be statistically significant.

### Quantitative real time PCR analysis

Quantitative real time PCR (qRT-PCR) was performed using a LightCycler 96 Sequence Detection system (Roche Co., Ltd., Basel, Switzerland). cDNA was synthesized from rice RNA using PrimeScript RT reagent Kit with gDNA Eraser (TaKaRa Bio Inc., Otsu, Shiga, Japan). Primers were designed on NCBI database (https://www.ncbi.nlm.nih.gov/) and synthesized from Sangon Biotech (Co., Ltd., Shanghai, China). The primers used in this work were listed in Table S10. 18s rRNA was quantified as an internal control and the relative expression of target gene mRNA was calculated using the 2^−ΔΔCT^ method.

### Statistical analysis

All of parameters were repeated thrice. The data were expressed as mean ± standard deviation (SD). Two-tailed Student’s t-test was performed to test the effects of Na_2_CO_3_ treatment. SPSS 16.0. and GraphPad Prism (Version 8) performed all statistical analysis. *P* < 0.05 was defined as statistically significant.

## Supplementary Information

**Additional file 1: Table S1.** Sequencing statistics for rice seedling shoots. **Table S2.** Statistical analysis of clean data mapped to reference genome. **Fig. S1** Validation of the expression level of genes from RNA sequencing (RNA-seq) using qRT-PCR. Comparison of fold change (FC) was done by scatter plots using log_2_(FC) values obtained from RNA-seq and qRT-PCR. Blue dots indicate the DEGs in the Na_2_CO_3_-treated (N+) versus -untreated (N-) rice seedling shoots at day 1. Pink dots indicate the DEGs in the N+ versus N- rice seedling shoots at day 5. **Table S3.** Comparison of RNA-seq data and PCR data. **Fig. S2 A** Venn diagram of DEGs overlapping across the 1 d and 5 d of Na_2_CO_3_-treated (N+) versus -untreated (N-) rice seedling shoots. **B** Genes with opposite expression trends in Na_2_CO_3_-treated (N+) versus -untreated (N-) rice seedling shoots in 1 d and 5 d. **Table S6.** Common up-regulated genes in day 1 and day 5. **Table S7.** Common down-regulated genes in day 1 and day 5. **Table S8.** Common significantly enriched GO terms in day 1 and day 5. **Table S9.** Common significantly enriched KEGG pathways in day 1 and day 5. **Table S10.** Primers used for theqRT-PCR.

**Additional file 2: Table S4.** List of DEGs at day 1. **Table S5.** List of DEGs at day 5.

## Data Availability

The raw sequences were deposited into Sequence Read Archive database with the BioProject under the accession number PRJNA895747 (https://www.ncbi.nlm.nih.gov/bioproject/PRJNA895747).
