# Correction: Application of RNA sequencing to understand the response of rice seedlings to salt-alkali stress

**DOI:** 10.1186/s12864-023-09152-4

**Published:** 2023-02-03

**Authors:** Xiaoning Ren, Jiahui Fan, Xin Li, Yu Shan, Lanlan Wang, Lianju Ma, Yueying Li, Xuemei Li

**Affiliations:** College of Life Science, Shenyang Normal University, Shenyang, 110034 China

**Correction: BMC Genomics 24, 21 (2023)**

**https://doi.org/10.1186/s12864-023-09121-x**

Following the publication of the original article [1], the author reported that the symbol for equal contribution (^†^) was missing. Xiaoning Ren and Jiahui Fan contributed equally to this article. The character has been added.

The original article [1] has been updated.

## References

[1] Ren X, Fan J, Li X (2023). Application of RNA sequencing to understand the response of rice seedlings to salt-alkali stress. BMC Genomics..

