# Correction to: Application of RNA sequencing to understand the response of rice seedlings to salt-alkali stress

**DOI:** 10.1186/s12864-023-09357-7

**Published:** 2023-05-12

**Authors:** Xiaoning Ren, Jiahui Fan, Xin Li, Yu Shan, Lanlan Wang, Lianju Ma, Yueying Li, Xuemei Li

**Affiliations:** College of Life Science, Shenyang Normal University, Shenyang, 110034 China

**Correction to**: *BMC Genomics***24, 21 (2023).**

10.1186/s12864-023-09121-x

Following the publication of the original article [1], the authors reported that the 5th author was omitted from the author group. Lanlan Wang has been added to the author group and is presented correctly in this correction article.

The original article [1] has been updated.
