# Supplementary material for: Application of RNA sequencing to understand the response of rice seedlings to salt-alkali stress
Source: BMC Genomics. 2023 Jan 14;24:21. doi: 10.1186/s12864-023-09121-x (PMC9840837; doi:10.1186/s12864-023-09121-x)
Supplement: Supplementary file 2 — Additional file 2: Table S4. List of DEGs at day 1. Table S5. List of DEGs at day 5. [file 12864_2023_9121_MOESM2_ESM.docx]

**Table S1**

Sequencing statistics for rice seedling shoots

| Treatment time | Samples | Clean reads | Clean bases | GC content | %≥Q30 |
| --- | --- | --- | --- | --- | --- |
| Day 1 | N-1 | 25,526,561 | 7,625,099,206 | 52.51% | 94.26% |
|  | N-2 | 28,015,206 | 8,381,747,932 | 52.73% | 94.59% |
|  | N-3 | 23,683,043 | 7,093,441,102 | 52.85% | 94.09% |
|  | N+1 | 25,366,815 | 7,591,074,958 | 51.83% | 94.48% |
|  | N+2 | 20,692,370 | 6,196,932,466 | 51.66% | 94.16% |
|  | N+3 | 24,496,430 | 7,329,738,488 | 51.54% | 94.05% |
| Day 5 | N-1 | 25,242,781 | 7,543,121,222 | 51.29% | 94.16% |
|  | N-2 | 23,089,474 | 6,902,309,340 | 50.47% | 94.38% |
|  | N-3 | 22,490,937 | 6,718,324,032 | 50.48% | 94.43% |
|  | N+1 | 21,837,888 | 6,533,424,348 | 51.29% | 93.87% |
|  | N+2 | 28,318,468 | 8,469,271,514 | 51.62% | 94.34% |
|  | N+3 | 23,956,265 | 7,152,569,430 | 51.44% | 94.36% |

Note: N- indicates the rice shoots without Na_2_CO_3_ stress, N+ indicates the rice shoots with Na_2_CO_3_ stress.

**Table S2**

Statistical analysis of clean data mapped to reference genome

| Treatment time | Samples | Total reads | Mapped reads | Mapped ratio | Reads Map to '+' | Reads Map to '-' |
| --- | --- | --- | --- | --- | --- | --- |
| Day 1 | N-1 | 51,053,122 | 48,894,081 | 95.77% | 24,313,664 | 24,390,806 |
|  | N-2 | 56,030,412 | 53,713,667 | 95.87% | 26,690,257 | 26,785,412 |
|  | N-3 | 47,366,086 | 45,441,034 | 95.94% | 22,587,435 | 22,659,184 |
|  | N+1 | 50,733,630 | 48,597,900 | 95.79% | 24,180,310 | 24,250,238 |
|  | N+2 | 41,384,740 | 39,676,412 | 95.87% | 19,741,339 | 19,803,394 |
|  | N+3 | 48,992,860 | 46,845,711 | 95.62% | 23,291,737 | 23,375,380 |
| Day 5 | N-1 | 50,485,562 | 48,306,938 | 95.68% | 24,014,932 | 24,095,791 |
|  | N-2 | 46,178,948 | 44,164,059 | 95.64% | 21,964,237 | 22,030,304 |
|  | N-3 | 44,981,874 | 43,053,431 | 95.71% | 21,407,673 | 21,477,473 |
|  | N+1 | 43,675,776 | 41,597,173 | 95.24% | 20,671,106 | 20,754,170 |
|  | N+2 | 56,636,936 | 54,126,722 | 95.57% | 26,879,505 | 26,996,982 |
|  | N+3 | 47,912,530 | 45,839,933 | 95.67% | 22,801,551 | 22,869,609 |

Note: N- indicates the rice shoots without Na_2_CO_3_ stress, N+ indicates the rice shoots with Na_2_CO_3_ stress.

**
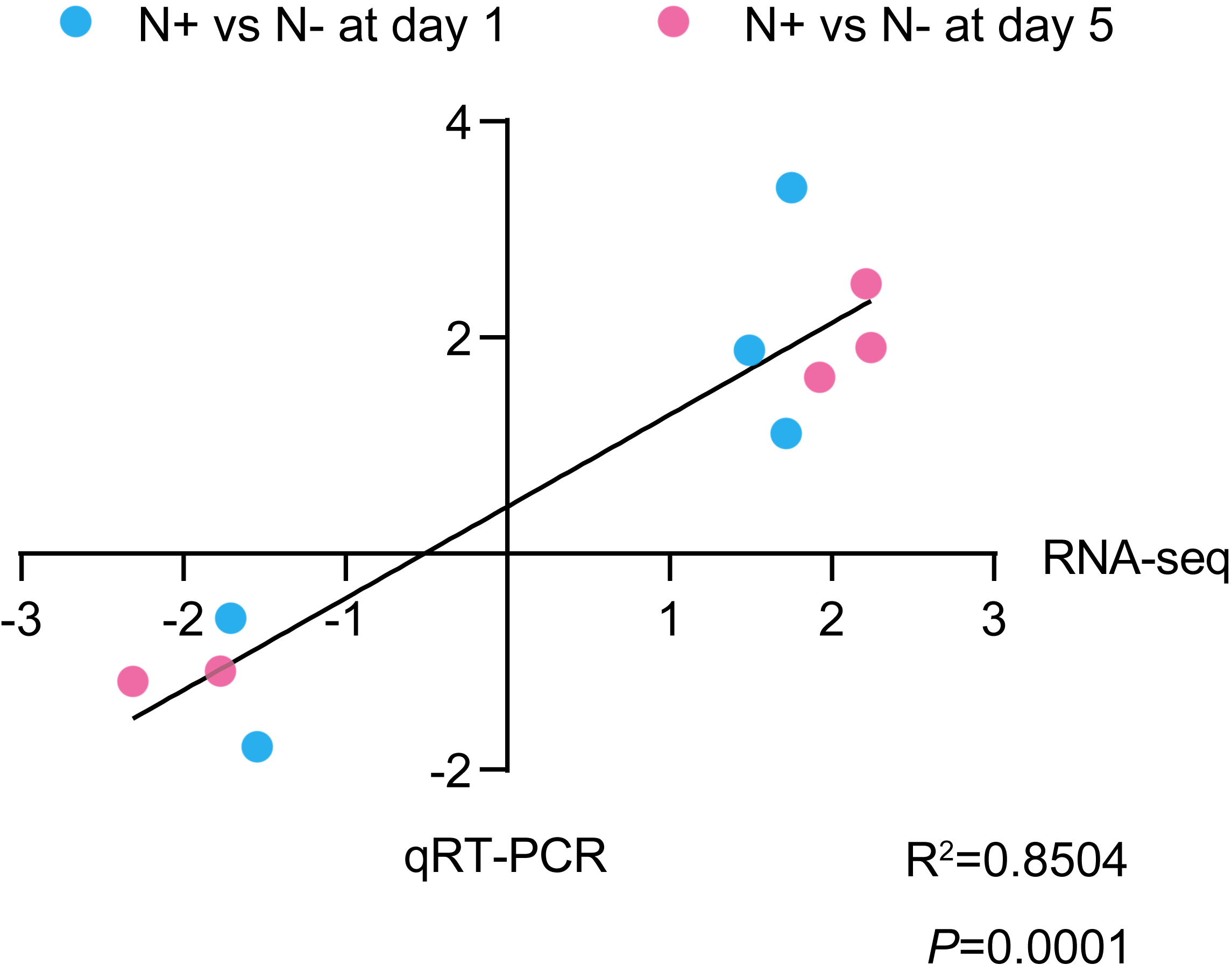
**

**Fig. S1** Validation of the expression level of genes from RNA sequencing (RNA-seq) using qRT-PCR. Comparison of fold change (FC) was done by scatter plots using log_2_(FC) values obtained from RNA-seq and qRT-PCR. Blue dots indicate the DEGs in the Na_2_CO_3_-treated (N+) versus -untreated (N-) rice seedling shoots at day 1. Pink dots indicate the DEGs in the N+ versus N- rice seedling shoots at day 5.

**Table S3**

Comparison of RNA-seq data and PCR data

| Treatment time | Gene ID | Log_2_(FC) of RNA-seq | Log_2_(FC) of qRT-PCR | P-value |
| --- | --- | --- | --- | --- |
| Day 1 | Os01g0858350 | 1.72 | 1.11 | 0.83 |
|  | Os04g0513400 | 1.75 | 3.39 |  |
|  | Os02g0669100 | 1.49 | 1.88 |  |
|  | Os01g0720500 | -1.71 | -0.60 |  |
|  | Os04g0683700 | -1.54 | -1.79 |  |
| Day 5 | Os10g0416500 | 2.24 | 1.91 | 0.72 |
|  | Os11g0700900 | 1.93 | 1.63 |  |
|  | Os03g0856700 | 2.21 | 2.50 |  |
|  | Os03g0180800 | -1.77 | -1.09 |  |
|  | Os04g0412300 | -2.31 | -1.18 |  |


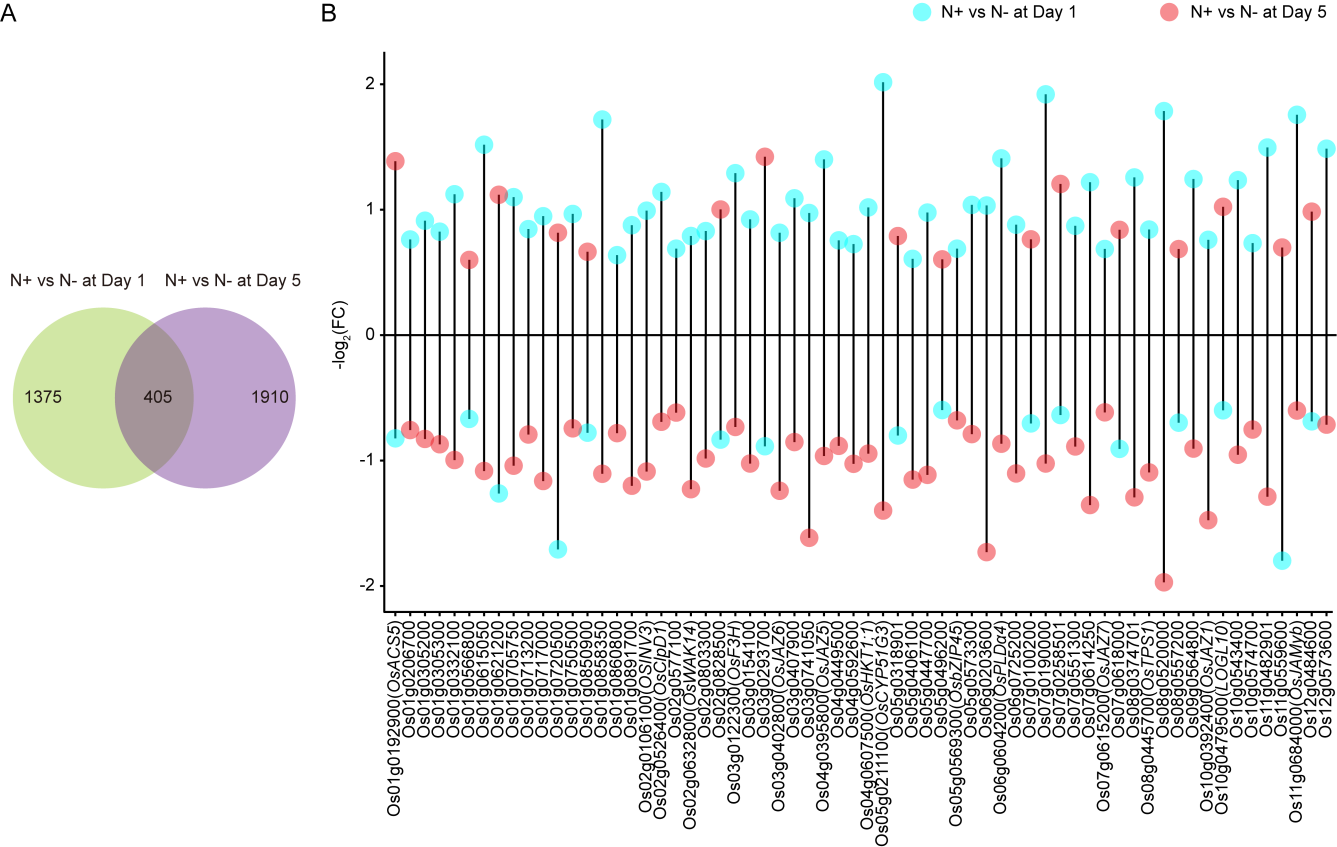
**Fig. S2 A** Venn diagram of DEGs overlapping across the 1 d and 5 d of Na_2_CO_3_-treated (N+) versus -untreated (N-) rice seedling shoots. **B** Genes with opposite expression trends in Na_2_CO_3_-treated (N+) versus -untreated (N-) rice seedling shoots in 1 d and 5 d.

**Table S6**

Common up-regulated genes in day 1 and day 5

| Gene ID | Log_2_(FC) at day 1 | Log_2_(FC) at day 5 |
| --- | --- | --- |
| Os01g0119000  Os01g0124200  Os01g0124401  Os01g0124650  Os01g0138900  Os01g0155800  Os01g0160800  Os01g0176200  Os01g0192300  Os01g0218100  Os01g0224000  Os01g0228500  Os01g0348900  Os01g0369700  Os01g0511000  Os01g0511100  Os01g0606900  Os01g0615100  Os01g0644000  Os01g0664500  Os01g0702000  Os01g0711000  Os01g0733200  Os01g0740650  Os01g0767600  Os01g0794400  Os01g0854000  Os01g0859300  Os01g0872100  Os01g0916600  Os01g0926300  Os01g0976300  Os02g0102900  Os02g0139000  Os02g0574500  Os02g0716800  Os02g0730000  Os02g0753800  Os02g0755900  Os02g0774100  Os02g0783625  Os02g0783700  Os02g0813350  Os03g0106200  Os03g0146300  Os03g0218400  Os03g0235000  Os03g0275300  Os03g0287100  Os03g0298300  Os03g0350100  Os03g0643250  Os03g0679700  Os03g0689300  Os03g0693700  Os03g0693900  Os03g0694000  Os03g0700400  Os03g0712800  Os03g0790500  Os03g0797400  Os03g0826800  Os03g0835400  Os04g0136700  Os04g0209200  Os04g0223500  Os04g0266900  Os04g0446500  Os04g0469500  Os04g0476600  Os04g0511200  Os04g0513400  Os04g0573000  Os04g0635100  Os04g0650800  Os04g0675400  Os04g0676800  Os04g0687100  Os05g0115100  Os05g0195101  Os05g0195700  Os05g0244700  Os05g0247100  Os05g0355400  Os05g0475400  Os05g0541400  Os05g0542900  Os05g0568100  Os05g0580000  Os05g0595300  Os06g0129900  Os06g0160700  Os06g0181100  Os06g0246500  Os06g0250600  Os06g0347966  Os06g0474800  Os06g0493100  Os06g0586000  Os06g0599200  Os06g0649600  Os06g0668200  Os06g0697200  Os06g0698300  Os07g0147900  Os07g0187300  Os07g0188700  Os07g0195300  Os07g0519600  Os07g0529600  Os07g0539300  Os07g0550500  Os07g0592600  Os07g0633200  Os07g0656200  Os07g0683600  Os07g0684800  Os07g0693500  Os08g0112300  Os08g0126300  Os08g0191700  Os08g0335500  Os08g0425800  Os08g0434632  Os08g0458200  Os08g0544400  Os09g0109600  Os09g0298200  Os09g0344500  Os09g0365300  Os09g0426100  Os09g0467200  Os09g0472100  Os09g0472900  Os09g0491740  Os10g0109600  Os10g0118200  Os10g0360100  Os10g0389300  Os10g0445400  Os10g0491000  Os10g0517500  Os10g0521000  Os10g0525500  Os10g0552400  Os10g0558700  Os10g0565150  Os10g0569400  Os10g0569600  Os10g0576600  Os11g0125900  Os11g0149400  Os11g0199200  Os11g0444700  Os11g0494100  Os11g0592000  Os11g0592200  Os11g0594200  Os11g0634200  Os11g0700900  Os11g0704500  Os12g0123500  Os12g0123800  Os12g0133100  Os12g0137100  Os12g0183100  Os12g0210500  Os12g0268000  Os12g0283100  Os12g0428000  Os12g0508200  Os12g0555200  Os12g0555500  Os12g0564100  Os12g0609800 | 1.04  1.27  2.01  1.76  1.08  1.21  1.20  1.05  1.36  1.23  0.89  1.27  1.63  0.76  1.06  1.52  2.41  1.52  1.07  0.72  1.23  0.88  1.45  0.86  0.78  0.98  0.76  1.33  0.86  0.96  0.65  1.44  0.71  1.54  0.97  1.97  1.49  0.84  1.42  0.98  1.36  1.44  0.59  0.77  1.23  1.20  1.23  0.73  0.75  0.82  0.60  1.32  1.31  0.78  1.65  0.84  0.85  1.56  1.46  1.69  1.29  1.76  0.69  0.99  0.80  1.94  1.16  0.71  0.93  1.57  1.99  1.75  0.82  0.67  0.68  2.11  2.49  0.59  1.27  0.80  1.08  0.96  0.91  0.65  1.19  1.46  0.99  1.18  1.47  1.02  1.17  1.36  0.79  1.84  0.60  0.85  1.84  1.08  1.16  0.82  0.92  1.37  1.57  2.06  0.71  0.66  1.01  1.51  0.77  1.85  1.10  0.77  1.14  1.18  0.98  1.25  1.61  0.91  1.15  0.59  0.89  1.58  1.06  1.14  0.97  1.54  1.44  1.29  1.74  0.86  1.41  0.84  1.22  1.50  0.99  1.11  2.64  1.26  1.08  1.47  1.24  0.93  2.04  0.73  1.47  0.96  0.98  1.63  1.60  1.06  0.99  0.81  0.74  0.78  1.21  1.45  1.19  0.80  1.73  1.03  0.63  0.75  1.67  0.71  0.68  0.64  1.01  1.15  0.78  0.85  1.21  1.23  1.41  1.52  0.69 | 1.01  0.65  0.71  0.79  0.78  1.10  1.21  1.23  0.64  1.18  0.74  0.85  1.20  1.11  1.50  1.29  0.63  0.83  0.68  0.59  0.91  0.70  0.68  0.60  0.73  0.70  0.71  1.04  1.00  0.76  0.65  1.16  0.93  0.95  0.62  3.00  1.32  0.85  1.93  0.72  0.94  1.13  0.75  1.03  0.59  0.66  0.96  0.62  0.66  0.61  0.79  1.18  0.65  1.12  2.06  2.06  1.94  1.24  2.93  0.68  1.29  0.98  0.95  1.41  0.78  1.96  0.94  0.73  0.80  0.98  0.70  0.89  1.23  1.57  0.75  1.60  1.10  0.78  0.94  0.82  0.64  1.05  0.99  0.73  0.89  1.22  1.06  0.74  1.16  0.72  0.62  0.79  0.97  1.25  1.15  0.66  0.99  1.63  0.62  1.49  0.86  1.41  2.12  1.09  0.63  0.60  1.96  0.99  0.83  1.11  0.92  0.88  1.15  1.05  1.34  0.73  0.81  0.73  1.41  0.71  0.94  1.54  0.73  1.46  0.64  1.67  0.98  1.40  1.73  0.65  0.98  0.70  0.81  1.75  0.99  0.97  2.63  1.54  0.98  0.62  1.26  1.32  1.54  0.88  1.05  0.78  0.70  1.69  1.83  2.10  1.12  1.44  0.62  0.90  0.90  2.77  2.13  0.59  0.59  1.93  0.70  1.21  0.66  0.74  0.81  0.69  0.63  0.76  1.27  1.30  0.79  1.11  1.34  0.90  0.59 |

**Table S7**

Common down-regulated genes in day 1 and day 5

| Gene ID | Log_2_(FC) at day 1 | Log_2_(FC) at day 5 |
| --- | --- | --- |
| Oryza_sativa_newGene_10205  Oryza_sativa_newGene_1324  Oryza_sativa_newGene_1657  Oryza_sativa_newGene_1879  Oryza_sativa_newGene_3125  Oryza_sativa_newGene_3730  Oryza_sativa_newGene_3969  Oryza_sativa_newGene_4426  Oryza_sativa_newGene_4430  Oryza_sativa_newGene_5211  Oryza_sativa_newGene_5278  Oryza_sativa_newGene_5713  Oryza_sativa_newGene_6000  Oryza_sativa_newGene_6098  Oryza_sativa_newGene_647  Oryza_sativa_newGene_721  Oryza_sativa_newGene_8379  Oryza_sativa_newGene_9276  Oryza_sativa_newGene_9785  Os01g0134700  Os01g0172600  Os01g0186900  Os01g0276900  Os01g0278050  Os01g0281100  Os01g0357100  Os01g0384800  Os01g0557500  Os01g0582600  Os01g0585300  Os01g0702700  Os01g0714600  Os01g0719700  Os01g0725000  Os01g0739500  Os01g0757200  Os01g0817000  Os01g0819700  Os01g0826400  Os01g0874300  Os01g0902100  Os01g0905200  Os01g0905300  Os01g0934100  Os01g0934400  Os01g0940000  Os01g0949300  Os02g0109200  Os02g0130100  Os02g0134400  Os02g0182700  Os02g0251800  Os02g0438601  Os02g0522300  Os02g0540700  Os02g0621600  Os02g0627700  Os02g0665600  Os02g0673500  Os02g0677300  Os02g0703600  Os02g0716600  Os02g0759400  Os02g0765900  Os02g0770800  Os03g0133000  Os03g0161900  Os03g0187550  Os03g0190300  Os03g0226200  Os03g0300900  Os03g0310800  Os03g0319400  Os03g0437200  Os03g0575700  Os03g0609500  Os03g0664800  Os03g0710000  Os03g0723800  Os03g0764100  Os03g0773000  Os03g0805600  Os03g0826200  Os04g0116600  Os04g0349600  Os04g0418500  Os04g0492800  Os04g0518400  Os04g0526800  Os04g0556500  Os04g0571600  Os04g0578000  Os04g0586500  Os04g0604300  Os04g0610400  Os04g0640850  Os04g0650700  Os04g0667600  Os04g0669475  Os04g0683700  Os05g0170950  Os05g0176700  Os05g0199100  Os05g0243200  Os05g0324700  Os05g0424000  Os05g0453300  Os05g0468900  Os05g0476700  Os05g0495300  Os05g0548900  Os05g0555300  Os06g0102100  Os06g0239200  Os06g0487620  Os06g0649000  Os06g0661800  Os06g0662200  Os06g0666400  Os06g0704200  Os07g0133500  Os07g0474700  Os07g0523400  Os07g0523600  Os07g0523965  Os07g0573900  Os07g0680600  Os07g0685500  Os07g0686500  Os08g0173300  Os08g0185701  Os08g0249000  Os08g0442350  Os08g0448000  Os08g0457400  Os08g0460000  Os08g0465700  Os08g0468700  Os08g0468801  Os08g0482600  Os08g0544800  Os09g0135800  Os09g0241100  Os09g0328600  Os09g0400500  Os09g0445500  Os09g0513500  Os09g0522000  Os09g0562750  Os10g0124100  Os10g0124150  Os10g0320100  Os10g0499400  Os10g0510500  Os10g0519400  Os11g0118400  Os11g0118500  Os11g0143200  Os11g0655900  Os11g0656000  Os12g0139300  Os12g0164450  Os12g0181500  Os12g0186600  Os12g0538600  Os12g0538700 | -0.63  -0.86  -0.75  -0.95  -1.28  -0.67  -0.92  -1.29  -1.04  -0.66  -1.15  -0.87  -1.08  -1.06  -0.85  -0.71  -0.82  -0.98  -1.22  -1.09  -0.62  -1.23  -1.28  -1.11  -1.54  -1.39  -1.03  -0.71  -1.14  -0.67  -1.14  -1.01  -0.66  -1.02  -1.12  -0.67  -1.01  -0.83  -1.68  -1.17  -0.63  -1.93  -1.72  -1.21  -0.83  -1.11  -1.52  -0.89  -0.74  -0.95  -0.68  -1.11  -1.40  -0.59  -1.21  -0.83  -0.88  -0.91  -0.96  -1.70  -1.13  -1.51  -1.59  -1.38  -1.29  -0.96  -1.00  -1.11  -1.31  -1.18  -0.63  -1.42  -1.32  -1.13  -1.35  -2.15  -0.90  -1.16  -1.06  -1.03  -1.22  -0.62  -0.96  -0.87  -0.85  -1.03  -0.76  -0.96  -1.21  -0.81  -1.69  -0.96  -0.88  -1.15  -1.81  -1.12  -1.23  -0.91  -0.81  -1.54  -0.68  -0.84  -1.38  -0.59  -1.46  -0.72  -0.79  -1.17  -1.16  -0.75  -0.77  -0.90  -0.90  -1.85  -1.50  -1.14  -0.65  -1.40  -0.67  -0.63  -1.00  -0.64  -1.31  -1.13  -0.94  -1.14  -1.35  -0.68  -0.70  -1.46  -0.80  -1.04  -0.68  -0.93  -1.69  -1.27  -0.80  -1.08  -1.28  -1.39  -0.76  -0.90  -0.73  -1.10  -1.20  -1.06  -1.04  -1.14  -1.77  -0.84  -0.82  -1.33  -1.12  -0.86  -1.15  -0.80  -1.08  -1.43  -2.62  -1.41  -0.96  -1.06  -0.89  -0.91  -1.71  -1.01 | -1.25  -0.99  -0.69  -1.44  -1.20  -0.61  -0.75  -1.14  -1.20  -1.36  -1.02  -1.10  -0.79  -0.63  -0.83  -0.91  -1.09  -2.15  -1.02  -0.66  -0.66  -1.38  -0.92  -0.90  -0.64  -1.35  -0.62  -0.72  -1.20  -0.61  -1.37  -1.22  -1.08  -0.95  -1.00  -0.89  -0.70  -0.71  -1.35  -0.68  -0.79  -1.67  -1.33  -0.61  -1.33  -1.19  -0.90  -0.80  -0.67  -0.66  -0.63  -0.97  -0.61  -0.68  -1.03  -0.95  -0.72  -0.69  -0.87  -1.70  -0.76  -1.39  -1.91  -1.29  -1.41  -0.99  -0.98  -1.01  -1.15  -2.42  -0.68  -1.29  -1.10  -1.60  -1.25  -1.05  -0.73  -0.95  -0.82  -0.73  -0.97  -0.67  -1.35  -0.75  -1.04  -0.74  -0.82  -1.01  -0.99  -0.93  -1.71  -1.38  -0.96  -2.42  -0.94  -0.62  -1.98  -0.70  -0.84  -0.93  -0.79  -0.83  -0.68  -0.63  -0.92  -1.00  -1.22  -1.55  -1.20  -0.80  -0.68  -1.21  -0.92  -1.19  -0.76  -1.40  -0.71  -1.09  -0.74  -0.83  -1.37  -0.70  -0.68  -0.92  -0.65  -1.03  -1.40  -0.76  -0.88  -1.63  -0.86  -1.03  -0.72  -1.13  -1.67  -0.61  -0.78  -0.98  -0.81  -1.63  -0.81  -1.11  -0.62  -0.93  -0.73  -0.92  -1.16  -1.20  -1.10  -1.03  -1.08  -1.77  -0.84  -1.06  -1.01  -0.61  -1.04  -1.14  -0.83  -1.23  -0.75  -1.61  -0.70  -1.07  -1.05  -0.88 |

**Table S8**

Common significantly enriched GO terms in day 1 and day 5

|  | GO ID | Description | P-value at day 1 | P-value at day 5 |
| --- | --- | --- | --- | --- |
| Biological Progress | GO:0055114 | oxidation-reduction process | <0.01 | <0.01 |
|  | GO:0042128 | nitrate assimilation | <0.01 | 0.01 |
|  | GO:0018298 | protein-chromophore linkage | <0.01 | 0.04 |
|  | GO:0006809 | nitric oxide biosynthetic process | <0.01 | 0.01 |
|  | GO:1903507 | negative regulation of nucleic acid-templated transcription | <0.01 | <0.01 |
|  | GO:0009228 | thiamine biosynthetic process | <0.01 | <0.01 |
|  | GO:2000022 | regulation of jasmonic acid mediated signaling pathway | <0.01 | <0.01 |
|  | GO:0031347 | regulation of defense response | <0.01 | <0.01 |
|  | GO:0042218 | 1-aminocyclopropane-1-carboxylate biosynthetic process | <0.01 | 0.03 |
|  | GO:0009911 | positive regulation of flower development | <0.01 | 0.03 |
|  | GO:0009611 | response to wounding | <0.01 | <0.01 |
|  | GO:0010951 | negative regulation of endopeptidase activity | <0.01 | 0.02 |
|  | GO:0005992 | trehalose biosynthetic process | <0.01 | 0.05 |
|  | GO:0048657 | anther wall tapetum cell differentiation | 0.02 | <0.01 |
|  | GO:0000272 | polysaccharide catabolic process | 0.02 | <0.01 |
|  | GO:0016132 | brassinosteroid biosynthetic process | 0.02 | <0.01 |
|  | GO:0016125 | sterol metabolic process | 0.03 | <0.01 |
|  | GO:0006355 | regulation of transcription, DNA-templated | 0.03 | 0.03 |
|  | GO:0010268 | brassinosteroid homeostasis | 0.03 | <0.01 |
|  | GO:0005975 | carbohydrate metabolic process | 0.04 | <0.01 |
|  | GO:0006559 | L-phenylalanine catabolic process | 0.04 | 0.02 |
|  | GO:0034219 | carbohydrate transmembrane transport | 0.05 | <0.01 |
|  | GO:0006032 | chitin catabolic process | 0.05 | <0.01 |
| Cellular Component | GO:0009573 | chloroplast ribulose bisphosphate carboxylase complex | <0.01 | 0.05 |
|  | GO:0048046 | apoplast | <0.01 | 0.03 |
| Molecular Function | GO:0050464 | nitrate reductase (NADPH) activity | <0.01 | 0.01 |
|  | GO:0009703 | nitrate reductase (NADH) activity | <0.01 | 0.03 |
|  | GO:0043546 | molybdopterin cofactor binding | <0.01 | 0.02 |
|  | GO:0003714 | transcription corepressor activity | <0.01 | <0.01 |
|  | GO:0050162 | oxalate oxidase activity | <0.01 | <0.01 |
|  | GO:0030151 | molybdenum ion binding | <0.01 | 0.01 |
|  | GO:0048307 | ferredoxin-nitrite reductase activity | <0.01 | 0.03 |
|  | GO:0004497 | monooxygenase activity | <0.01 | <0.01 |
|  | GO:0020037 | heme binding | <0.01 | <0.01 |
|  | GO:0016847 | 1-aminocyclopropane-1-carboxylate synthase activity | 0.01 | 0.02 |
|  | GO:0005506 | iron ion binding | 0.01 | <0.01 |
|  | GO:0009055 | electron transfer activity | 0.04 | 0.01 |
|  | GO:0004568 | chitinase activity | 0.04 | <0.01 |
|  | GO:0008083 | growth factor activity | 0.05 | 0.01 |

**Table S9**

Common significantly enriched KEGG pathways in day 1 and day 5

| ID | Description | P-value at day 1 | P-value at day 5 |
| --- | --- | --- | --- |
| ko00910 | Nitrogen metabolism | <0.01 | 0.02 |
| ko00710 | Carbon fixation in photosynthetic organisms | <0.01 | <0.01 |
| ko00250 | Alanine, aspartate and glutamate metabolism | <0.01 | <0.01 |
| ko01200 | Carbon metabolism | 0.01 | <0.01 |
| ko00770 | Pantothenate and CoA biosynthesis | 0.02 | 0.03 |
| ko00905 | Brassinosteroid biosynthesis | 0.03 | 0.01 |
| ko00500 | Starch and sucrose metabolism | 0.04 | 0.03 |
| Ko00730 | Thiamine metabolism | 0.05 | <0.01 |

**Table S10**

Primers used for the qRT-PCR

| Gene ID | Primer sequence |
| --- | --- |
| Os01g0858350 | F: 5’-GACGGTGCCCATGCAGATAA-3’ |
|  | R: 5’-ACCTCTGGGTGATCGGAGAG-3’ |
| Os04g0513400 | F: 5’-GCGCATACTTGGACGACAAC-3’ |
|  | R: 5’-CGGCCTCTTAAAACAAGCACTG-3’ |
| Os02g0669100 | F: 5’-AAACTGCCCGGTTACCACAA-3’ |
|  | R: 5’-GCAGTGCAGAAAAAGCACCA-3’ |
| Os01g0720500 | F: 5’-TGTTCGGGTTCTTCGTCCAG-3’ |
|  | R: 5’-TACGCGGAAAGAGGAGAACG-3’ |
| Os04g0683700 | F: 5’-CTGCCCACCAAGGCGTAATA-3’ |
|  | R: 5’-TACGAAAGGACGGGAAACCG-3’ |
| 18s rRNA | F: 5’-ATGATAACTCGACGGATCGC-3’ |
|  | R: 5’-CTTGGATGTGGTAGCCGTTT-3’ |
